# Different Impact of Metabolic Syndrome on the Risk of Incidence of the Peripheral Artery Disease and the Venous Thromboembolism: A Nationwide Longitudinal Cohort Study in South Korea

**DOI:** 10.31083/j.rcm2404113

**Published:** 2023-04-17

**Authors:** Myung Soo Park, Jong Sun Ok, JiDong Sung, Duk-Kyung Kim, Seong Woo Han, Tae-Eun Kim, Bum Sung Kim, Hyun-Joong Kim, Sung Hea Kim, Hyeongsu Kim

**Affiliations:** ^1^Division of Cardiology, Dongtan Sacred Heart Hospital, Hallym University College of Medicine, 18450 Hwaseong, Republic of Korea; ^2^Department of Nursing, College of Nursing, Konkuk University, 27478 Chungju, Republic of Korea; ^3^Division of Cardiology, Department of Medicine, Heart Vascular Stroke Institute, Samsung Medical Center, Sungkyunkwan University School of Medicine, 06351 Seoul, Republic of Korea; ^4^Division of Cardiology, Department of Medicine, Samsung Changwon Hospital, Sungkyunkwan University School of Medicine, 51353 Changwon, Republic of Korea; ^5^Department of Clinical Pharmacology, Konkuk University Medical Center, 05030 Seoul, Republic of Korea; ^6^Division of Cardiology, Department of Internal Medicine, Konkuk University Medical Center, Konkuk University School of Medicine, 05030 Seoul, Republic of Korea; ^7^Department of Preventive Medicine, School of Medicine, Konkuk University, 05030 Seoul, Republic of Korea

**Keywords:** metabolic syndrome, peripheral arterial disease, venous thromboembolism, obesity

## Abstract

**Background:**

Limited data is available between metabolic syndrome (MetS) and the development of peripheral arterial disease (PAD) or venous thromboembolism (VTE) in the Asian population. We investigated the incidence of PAD and VTE according to the prevalence of MetS and evaluated the impact of individual components in MetS on the development of PAD and VTE using Korean national data.

**Methods:**

Data obtained from national health screening examinations of the Korean National Health Insurance Service from January 1, to December 31, 2009. In total, 9,927,538 participants, 7,830,602 participants were included in this study and the incidence rate of PAD and VTE was investigated retrospectively during a 7-year follow-up. Using the National Cholesterol Education Program Adult Treatment Panel III criteria, patients were placed into one of three groups depending on MetS component numbers: 0 (normal), 1–2 (Pre-MetS), or 3–5 (MetS).

**Results:**

The incidence rates of PAD and VTE in MetS were 2.25% and 0.71%, respectively. After multivariable adjustment, the risk of PAD was significantly associated with MetS (hazard ratio (HR) 1.45, 95% confidence interval (CI) 1.42–1.49), the risk of VTE was not associated with MetS (HR 1.01, 95% CI 0.96–1.05). When subgroup analyses were conducted according to MetS components, elevated fasting glucose (HR 1.26, 95% CI 1.23–1.27), abdominal obesity (HR 1.15, 95% CI 1.12–1.17), and elevated blood pressure (HR 1.13, 95% CI 1.12–1.15) were the most related to PAD. Abdominal obesity (HR 1.104, 95% CI 1.064–1.146) was associated with an increased risk of VTE.

**Conclusions:**

MetS was significantly associated with an increased incidence rate of PAD among the general Korean population. On the other hand, MetS was not associated with the VTE incidence rate. Of the MetS components, only abdominal obesity was a significant predictor of VTE.

## 1. Introduction

Metabolic syndrome (MetS) means that cardiovascular risk factors such as dyslipidemia, hypertension (HTN), obesity, and disturbed glucose metabolism appear as clustering [1]. The prevalence of MetS in adults is 20–30% worldwide [2]. According to the Korea National Health and Nutrition Examination Survey, the prevalence of adult MetS in Korea increased from 23.6% in 1998 to 31.3% in 2012 [3], and in the population aged 65 or older, the prevalence rate was raised up to 45% in 2018 [4]. MetS has been reported to be associated with cardiovascular disease (CVD) [5]. In particular, peripheral arterial disease (PAD) has been reported to be associated with MetS [6]. However, in the case of venous disease, there were differences in the results of the studies regarding the relationship with MetS according to the subtypes of venous disease [7].

There is limited data available between MetS and the development of PAD or venous thromboembolism (VTE) in the Asian population. Therefore, this study aimed to investigate the incidence rate of adult MetS in Korea with a 7-year retrospective follow-up based on the results of large National Health Examination data from the Korean National Health Insurance Service (NHIS) database, and to compare the incidence of PAD and VTE according to the presence of MetS. Also, this study investigated the effects of individual components of MetS on the development of PAD and VTE.

## 2. Materials and Methods

### 2.1 Study Population and Patient Definition

**Fig. 1.**
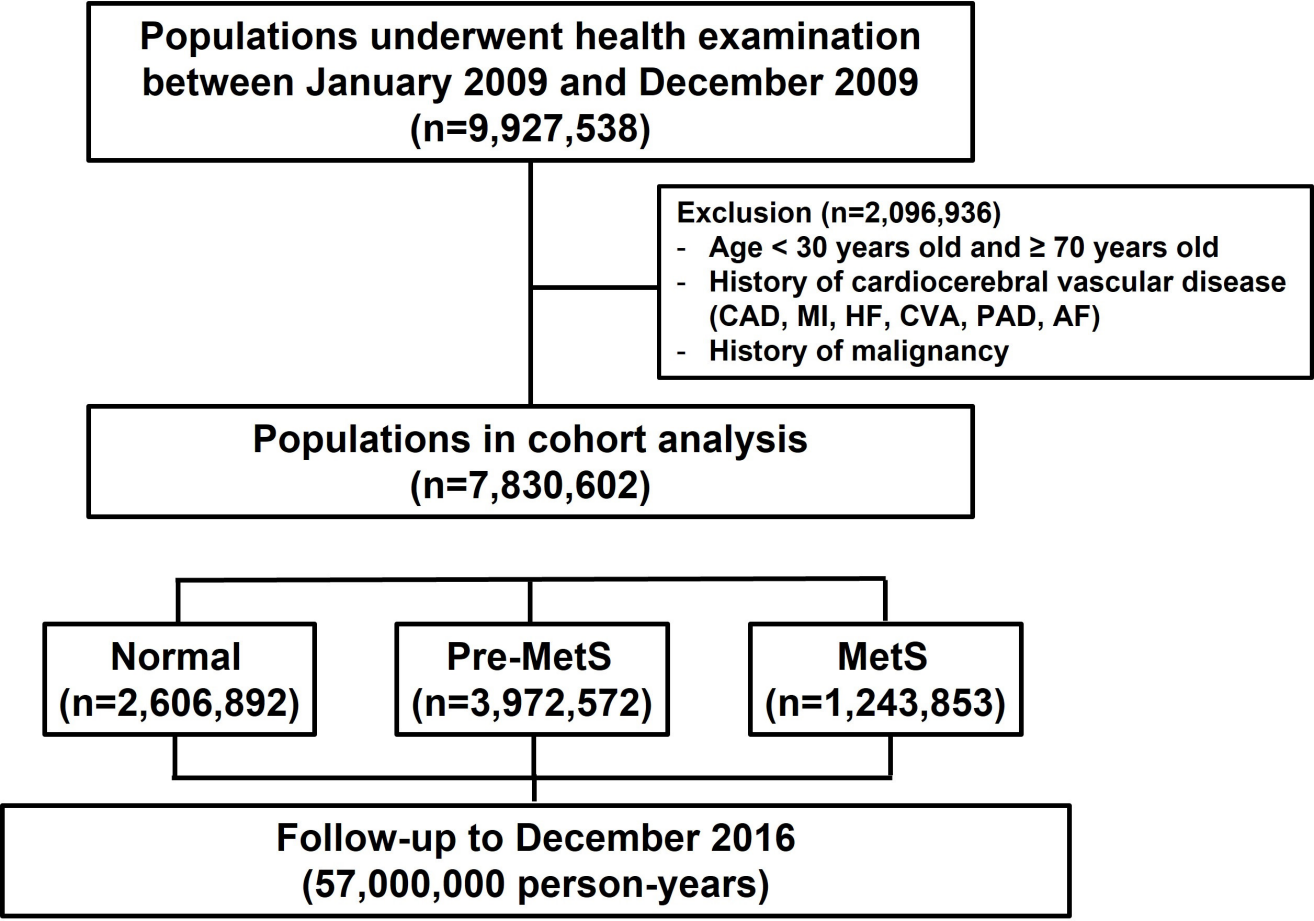
**Flow chart of the study**. Inclusion & exclusion criteria of the study population, and schematic study flow. CAD, coronary artery disease; MI, myocardial infarction; HF, heart failure; CVA, cerebrovascular accident; PAD, peripheral artery disease; AF, atrial fibrillation; MetS, metabolic syndrome.

Almost all South Koreans have national health insurance, and the NHIS provides various types of health services, including medical checkups for workers and regular medical checkups over the age of 40. Therefore, NHIS data includes people’s epidemiological characteristics, history of hospital service usage, and health examination data. The health examination section includes information on lifestyle through questionnaires, body measurement values, and blood test results. The Korean Industrial Safety and Health Law stipulates that employers provide health checkups to employees every year or two, and this data is also stored in the NHIS. The NHIS data classifies disease diagnosis through the International Classification of Disorders-Tenth revision (ICD-10) codes system. This study included 9,927,538 people’s data who received medical checkups in Korea in 2009, of which 2,096,936 were excluded according to the following exclusion criteria; (1) <30 years old, (2) ≥70 years old, (3) history of malignancy (ICD-10 codes C00.X-C99.X) and (4) history of cardiocerebral vascular disease, as following: coronary artery disease (procedure codes M6551-4), myocardial infarction (ICD-10 codes I21), heart failure (ICD-10 codes I42 or I50), cerebrovascular accident (ICD-10 codes I60.X-I609.X), and peripheral arterial disease (ICD-10 codes I73 or I74). The final study population consisted of 7,830,602 participants. Fig. 1 presents a schematic flow of the study population.

Patients with PAD were defined as a population with records of an outpatient visit or hospitalization in a tertiary hospital with the occurrence of the ICD-10 codes for PAD (I73 or I74) during the follow-up period. The VTE includes pulmonary thromboembolism (PTE) and deep vein thrombosis (DVT). Patients with PTE were defined as a population with records of hospitalization in a tertiary hospital with the occurrence of the ICD-10 code for PTE (I26) during the follow-up period. Patients with DVT were defined as a population with records of outpatient visits or hospitalization in a tertiary hospital with the occurrence of the ICD-10 code for DVT (I80) during the follow-up period. The tertiary hospitals are the medical institutions at the top of the medical delivery system implemented in Korea. They are selected by the Minister of Health and Welfare based on various indicators, and major hospitals across the country are included. This study was approved by the NHIS of Korea (No. NHIS-2020-1-537). This study complied with the regulations of the Institutional Review Board of Konkuk University Medical Center. The informed content was waived because NHIS data was used through a strict standard anonymization process.

### 2.2 Definition of Metabolic Syndrome (MetS)

The definition of MetS is generally in accordance with the modified criteria of the National Cholesterol Education Program (NCEP) Adult Treatment Panel III (ATP III) criteria. The diagnosis of MetS is possible if three or more of the following five components are applicable: (1) abdominal obesity (waist circumference (WC) ≥90 cm for men, ≥85 cm for women, modified criteria with Asian cutoffs for WC); (2) elevated blood pressure (BP) (systolic BP ≥130 mmHg or diastolic BP ≥85 mmHg or treatment of previously diagnosed HTN); (3) elevated fasting glucose (≥100 mg/dL or treatment of previously diagnosed diabetes mellitus (DM)); (4) high triglyceride (TG) (≥150 mg/dL or drug treatment for high TG); and (5) low high-density lipoprotein cholesterol (HDL-C) (<40 mg/dL for men, <50 mg/dL for women or drug treatment for low HDL-C). It is defined as pre-MetS if 1–2 of the five components are applicable, it is defined as normal if all are not applicable.

### 2.3 Statistical Analyses

Incidence rates were calculated as simple incidence rate and the number of events per 100,000 person-years. Incidence rates of PAD and VTE by sex, age group, and MetS status were compared using a chi-square test with Bonferroni’s correction for multiple testing as appropriate. We analyzed adjusted hazard ratios (HRs) for the incidence of PAD and VTE by use of Cox proportional hazards models with MetS status. The models were initially unadjusted. The first adjustments were made for sex, age, smoking status, and exercise status (Model 1). Model 2 was adjusted as Model 1, and plus for family history of hypertension (HTN), stroke, heart disease, and DM. Model 3 was adjusted as Model 2 and plus for body mass index (BMI), hemoglobin (Hb), creatinine (Cr), total cholesterol (TC), low-density lipoprotein cholesterol (LDL-C), and alanine aminotransferase (ALT). All tests were two or three-tailed, and *p *< 0.05 or *p *< 0.017 was considered statistically significant. All statistical calculations were performed using SAS version 9.1 (SAS Institute Inc., Cary, NC, USA).

## 3. Results

### 3.1 Overall Incidence Rate of PAD and VTE 

**Fig. 2.**
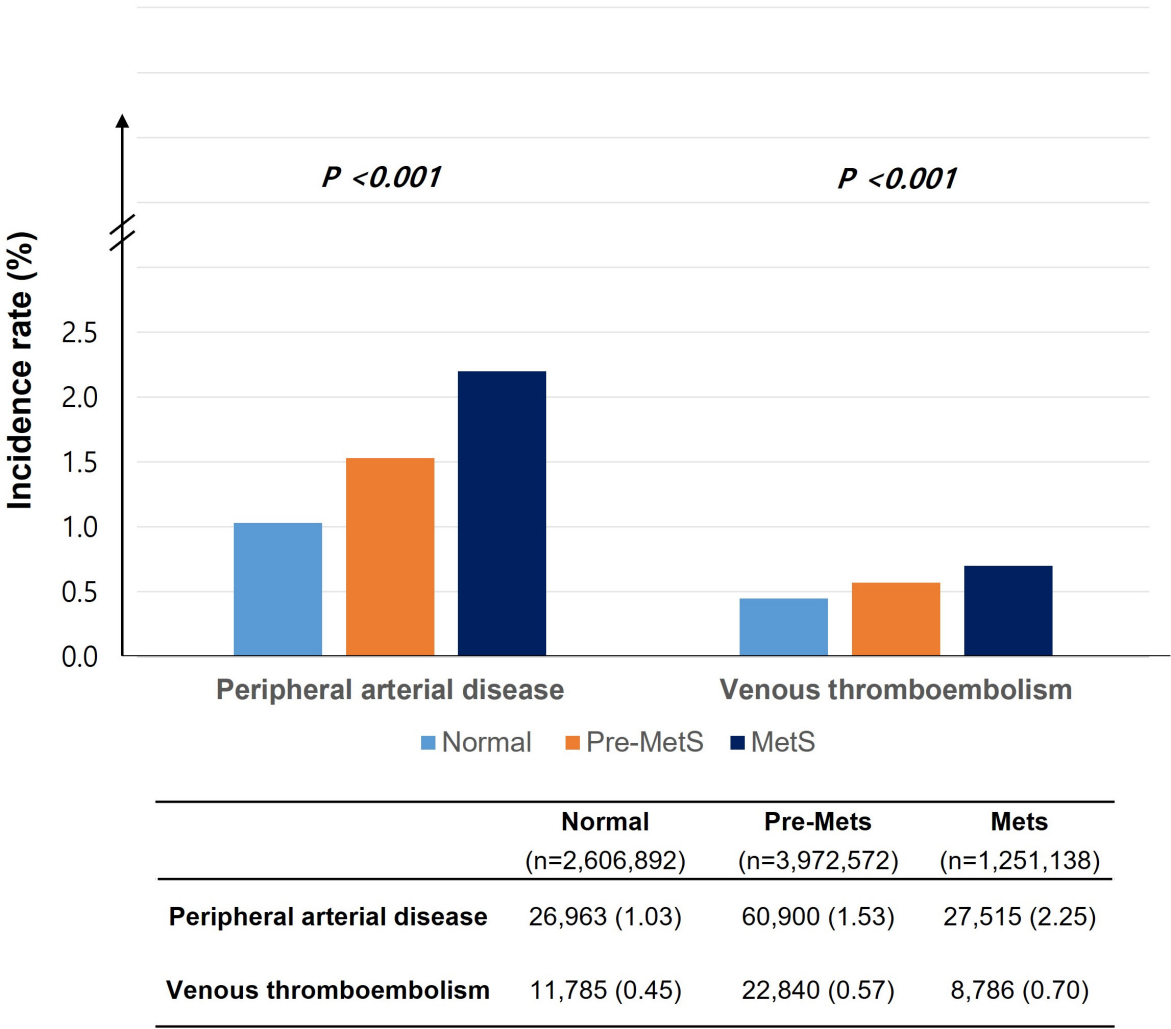
**The simple incidence rate of PAD and VTE according to MetS status**. The simple incidence rate before multivariate adjustment. PAD, peripheral artery disease; VTE, venous thromboembolism; MetS, metabolic syndrome.

A total of 7,830,602 participants were included in the cohort analysis. At the baseline, the prevalence of MetS was 1,251,138 (15.9%), and pre-MetS was present in 3,972,572 subjects (50.7%). During the total follow-up period of about 57,000,000 person-years, PAD occurred in 115,378 subjects (1.47%) and VTE occurred in 43,411 subjects (0.55%). The simple incidence rate of PAD according to MetS status was as follows: 26,963 (1.03%) in the normal group, 60,900 (1.53%) in the pre-MetS group, and 27,515 (2.25%) in the MetS group. The simple incidence rate of VTE according to MetS status was as follows: 11,785 (0.45%) in the normal group, 22,840 (0.57%) in the pre-MetS group, and 8786 (0.70%) in the MetS group (Fig. 2).

### 3.2 Association between MetS Status and Incidence Risk of PAD and VTE 

Tables 1,2 show the incidence rates (per 100,000 person-years) of PAD and VTE according to age group and the status of MetS in each gender population are shown in Tables 1,2. In the male gender, the incidence rates of PAD increased significantly according to MetS status in all age groups. The incidence rates of VTE increased according to aging, but there was no significant difference between MetS status. In the female gender, the incidence rates of PAD increased significantly according to MetS statuses in all age groups. The incidence rates of VTE increased according to aging. In the 50s and 60s of the female group, incidence rates of VTE increased significantly according to MetS status.

**Table 1.** **Incidence rate (per 100,000 person-years) of PAD and VTE in the male population according to age groups and status of metabolic syndrome**.

	MetS status	Age groups (years)			
		30–39	40–49	50–59	60–69
PAD	Normal	51.73	82.43	152.73	283.30
	Pre-Mets	59.92	95.74	197.92	365.76
	Mets	82.15	131.10	251.10	454.20
	*p*-value	<0.001	<0.001	<0.001	<0.001
VTE	Normal	41.21	50.17	64.07	120.67
	Pre-Mets	43.75	49.19	71.82	123.12
	Mets	48.96	52.26	74.21	132.04
	*p*-value	0.04	0.11	0.06	0.05

PAD, peripheral artery disease; VTE, venous thromboembolism; MetS, metabolic syndrome.

**Table 2.** **Incidence rate (per 100,000 person-years) of PAD and VTE in the female population according to age groups and status of metabolic syndrome**.

	Mets status	Age groups (years)			
		30–39	40–49	50–59	60–69
PAD	Normal	58.36	105.70	233.41	359.96
	Pre-Mets	65.98	131.29	271.76	435.84
	Mets	103.20	188.30	362.30	530.90
	*p*-value	<0.001	<0.001	<0.001	<0.001
VTE	Normal	36.01	49.57	81.44	114.53
	Pre-Mets	39.91	59.19	90.73	136.76
	Mets	46.64	57.32	115.98	164.07
	*p*-value	0.04	0.09	<0.001	<0.001

PAD, peripheral artery disease; VTE, venous thromboembolism; MetS, metabolic syndrome.

### 3.3 Risk of PAD and VTE According to MetS Status

Multivariable Cox regression analysis was performed to evaluate the association between MetS status and the incidence risk of PAD and VTE (Table 3). The non-adjusted HRs for PAD and VTE in MetS were 2.16 (95% CI 2.11–2.20), and 1.51 (1.45–1.56), respectively. After multivariable adjustment (Model 3), the risk of PAD was statistically significant in MetS (Adjusted HR 1.45, 95% CI 1.42–1.49). On the other hand, after multivariable adjustment (Model 3), the risk of VTE was not statistically significant in MetS (HR 1.01, 95% CI 0.96–1.05). A multivariable analysis revealed that over 40 years of age, smokers, and an increase in BMI were significant predictors of the increased risk of PAD and VTE.

**Table 3.** **Risk of developing peripheral arterial disease and venous thromboembolism according to the status of MetS: Cox proportional hazard model**.

		Peripheral arterial disease			
		Non-adjusted HR (95% CI)	Adjusted HR* (95% CI)		
			Model 1	Model 2	Model 3
MetS status					
	Normal	1	1	1	1
	Pre-MetS	1.48 (1.45–1.51)	1.21 (1.19–1.23)	1.20 (1.18–1.23)	1.18 (1.16–1.20)
	MetS	2.16 (2.11–2.20)	1.55 (1.52–1.58)	1.54 (1.50–1.57)	1.45 (1.42–1.49)
Sex					
	Female		1	1	1
	Male		0.73 (0.71–0.74)	0.73 (0.72–0.75)	0.74 (0.73–0.76)
Age group					
	30–39		1	1	1
	40–49		1.70 (1.64–1.76)	1.69 (1.63–1.75)	1.66 (1.60–1.72)
	50–59		3.52 (3.41–3.64)	3.50 (3.39–3.61)	3.43 (3.32–3.54)
	≥60		5.94 (5.75–6.13)	5.91 (5.73–6.10)	5.81 (5.62–6.00)
Smoking status					
	Non-smoke		1	1	1
	Ex-smoker		1.06 (1.04–1.09)	1.06 (1.03–1.09)	1.06 (1.03–1.09)
	Current smoker		1.08 (1.06–1.11)	1.08 (1.05–1.10)	1.09 (1.06–1.11)
Exercise					
	No exercise		1	1	1
	1–4 per week		0.99 (0.89–0.92)	0.90 (0.88–0.92)	0.90 (0.88–0.92)
	5 per week		0.92 (0.91–0.94)	0.92 (0.90–0.93)	0.92 (0.90–0.93)
Body mass index (kg/$m^{2}$)					1.02 (1.01–1.02)
Creatinine (mg/dL)					1.02 (1.01–1.02)
Total cholesterol (mg/dL)					1.00 (1.00–1.00)
LDL cholesterol (mg/dL)					1.00 (1.00–1.00)
		Venous thromboembolism			
		Non-adjusted HR (95% CI)	Adjusted HR* (95% CI)		
			Model 1	Model 2	Model 3
MetS status					
	Normal	1	1	1	1
	Pre-MetS	1.24 (1.21–1.28)	1.09 (1.06–1.12)	1.09 (1.06–1.12)	1.01 (0.98–1.04)
	MetS	1.51 (1.45–1.56)	1.20 (1.16–1.25)	1.20 (1.16–1.25)	1.01 (0.96–1.05)
Sex					
	Female		1	1	1
	Male		0.85 (0.82–0.88)	0.85 (0.82–0.88)	0.92 (0.88–0.96)
Age group					
	30–39		1	1	1
	40–49		1.20 (1.15–1.25)	1.20 (1.15–1.25)	1.16 (1.11–1.21)
	50–59		1.80 (1.73–1.88)	1.80 (1.73–1.88)	1.73 (1.66–1.80)
	≥60		2.82 (2.71–2.95)	2.82 (2.70–2.94)	2.74 (2.62–2.86)
Smoking status					
	Non-smoke		1	1	1
	Ex-smoker		0.99 (0.95–1.03)	0.99 (0.95–1.03)	0.99 (0.95–1.03)
	Current smoker		1.08 (1.05–1.12)	1.08 (1.05–1.12)	1.11 (1.07–1.16)
Exercise					
	No exercise		1	1	1
	1–4 per week		0.94 (0.91–0.97)	0.94 (0.91–0.97)	0.94 (0.91–0.97)
	5 per week		0.98 (0.95–1.01)	0.98 (0.95–1.01)	0.97 (0.94–1.00)
Body mass index (kg/$m^{2}$)					1.05 (1.04–1.05)
Creatinine (mg/dL)					0.98 (0.97–0.99)
Total cholesterol (mg/dL)					0.99 (0.99–1.00)
LDL cholesterol (mg/dL)					1.00 (0.99–1.00)

* Adjusted HR = adjusted for sex, age, smoking status, exercise, body mass index, creatinine, total cholesterol, low-density lipoprotein cholesterol. MetS, metabolic syndrome; HR, hazard ratio; CI, confidence intervals; BMI, body mass index; LDL, low-density lipoprotein.

### 3.4 Impact of MetS Component in the Incidence of PAD and VTE

The incidence risk of PAD and VTE according to the five components of MetS are shown in Table 4. Among the five components of MetS, elevated fasting glucose (HR 1.26, 95% CI 1.23–1.27), abdominal obesity (HR 1.15, 95% CI 1.12–1.17) and elevated blood pressure (HR 1.13, 95% CI 1.12–1.15) were the most related in PAD. Only abdominal obesity (HR 1.104, 95% CI 1.064–1.146) was associated with an increased risk of VTE.

**Table 4.** **Risk of developing peripheral arterial disease and venous thromboembolism according to individual components of MetS**.

	Peripheral arterial disease	Venous thromboembolism
	Adjusted HR* (95% CI)	Adjusted HR*(95% CI)
Abdominal obesity	1.15 (1.12–1.17)	1.10 (1.06–1.14)
Elevated blood pressure	1.13 (1.12–1.15)	1.00 (0.98–1.03)
Elevated fasting glucose	1.26 (1.23–1.27)	0.98 (0.96–1.01)
High triglyceride	1.05 (1.03–1.07)	0.96 (0.93–1.00)
Low HDL cholesterol	1.08 (1.06–1.10)	1.00 (0.97–1.04)

* Adjusted HR = adjusted for sex, age, smoking status, exercise, body mass index, creatinine, total cholesterol, low-density lipoprotein cholesterol. MetS, metabolic syndrome; HR, hazard ratio; CI, confidence intervals; HDL, high-density lipoprotein.

## 4. Discussion

In the present study, we investigated the incidence of PAD and VTE according to the prevalence of MetS and evaluated the impact of individual components in MetS on the development of PAD and VTE among the general Korean population using the NHIS database. During the 7-year follow-up period, the incidence rate of PAD was 1.47% in the general population and 2.25% in the population of MetS. The incidence rate of VTE was 0.55% in the general population and 0.70% in the population of MetS.

MetS has been reported to be associated with cardiovascular disease and various vascular diseases in the western population. In previous studies, the crude incidence rate of PAD with MetS was about 1.6–2.5%, and the relative risk of PAD increases 2–4 times when MetS is accompanied [8, 9]. In the present study cohorts, similar to previous studies, a similar incidence rate of PAD was observed and MetS was associated significantly with an increased incidence of PAD. PAD is thought to be related to the development of MetS because occlusive arterial disease is caused by atherosclerotic disease [10]. In this study, all five components of MetS were related to PAD development and especially, elevated fasting glucose (HR 1.26, 95% CI 1.23–1.27), abdominal obesity (HR 1.15, 95% CI 1.12–1.17) and elevated blood pressure (HR 1.13, 95% CI 1.12–1.15) were the most related among the five components of MetS. These results concord with those of prior studies by also showing that each component of MetS, such as blood pressure, blood sugar, and HDL-C, were associated with PAD [11].

In this study, the gender-based incidence of PAD was higher in women than in men, which is different from what is generally known. The primary cause of this unexpected result is that we excluded patients with coronary artery disease (CAD), PAD, and aged ≥70 years old from the analysis. The prevalence of PAD increases with age, and it is more prevalent in patients with CAD [12, 13]. Therefore, a significant number of high-risk patients with PAD may have been excluded from this cohort analysis. Through this patient selection process, the relationship between MetS and PAD incidence could be more clarified in this study. Still, on the other hand, it resulted in a distorted view of the epidemiological aspect.

The incidence rate of VTE is hard to conclude because it has a wide range according to the characteristics of the population. In general, the VTE incidence rate is known as 1–2 cases per 1000 people annually, and a lower incidence rate is reported in Asia [14, 15]. To date, limited studies are available between MetS and VTE and their association is inconclusive [16]. In this study cohort, the incidence rate of VTE has an increasing trend in the MetS groups, compared to the normal population. After applying multivariable-adjusted analysis, the incidence risk of VTE has no significant association with MetS. However, among the components of MetS, characteristically, abdominal obesity increased the risk of developing VTE (adjusted HR 1.10, 95% CI 1.06–1.14) after adjusting for various variables that may affect CVD. This result is consistent with previous studies on the relationship between VTE and MetS [17, 18]. In another study of risk factors for VTE from the Copenhagen City Heart Study, obesity and smoking were important risk factors for VTE whereas TC, HDL-C, LDL-C, and TG levels, and diabetes mellitus were not [19].

VTE, unlike PAD, is caused by the thrombus in the vein system. The thrombogenesis process is affected by abnormalities of blood flow, vessel integrity, and coagulation components [20]. Thrombogenic clinical conditions by various etiologies are major risk factors for VTE formation [21]. Among the factors of MetS, only abdominal obesity has been reported to be associated with the development of VTE. Although the mechanism is not clear, inflammatory reactions originating from adipose tissues are thought to increase thrombogenesis [22]. In this study, the association between the incidence of VTE and MetS was uncertain, but the association with abdominal obesity was confirmed, which is consistent with previous studies.

This study has the following limitations. First, this study used big data, but there are inherent limits to retrospective study design. Second, since cardiovascular and cerebrovascular disease patients were excluded, the association derived from this study is applied only to relatively healthy patients. Therefore, we cannot be sure that this association functions equally in cardio-cerebral vascular disease patients and high-risk populations. Third, this study used the ICD code to evaluate the incidence rate of PAD and VTE. The ICD code is a diagnosis code that can diagnose a disease with obvious symptoms, but screening for a disease with no symptoms is limited. Fourth, this study did not confirm the precedence of immobilization, which is a major risk factor for the development of VTE. However, our study had strengths in that it was a nationwide study with a large sample size and long-term follow-up periods. Thus, our results may be an important representation of the association between MetS and the risk of PAD or VTE among the general Korean population.

## 5. Conclusions

This nationwide longitudinal cohort demonstrated that MetS was significantly associated with an increased risk of PAD among the general Korean population and the five components of MetS were also associated with the risk of PAD. On the other hand, MetS as a cluster of risk factors was not associated with VTE risk. Of the MetS components, only abdominal obesity was a significant predictor of VTE.

## Data Availability

It is difficult to share the original data of this study because it is a property of the Korean National Health Insurance Service.
